# The Role of Angiogenesis in the Development of Proliferative Diabetic Retinopathy: Impact of Intravitreal Anti-VEGF Treatment

**DOI:** 10.1155/2012/728325

**Published:** 2012-04-11

**Authors:** Gemma Tremolada, Claudia Del Turco, Rosangela Lattanzio, Silvia Maestroni, Anna Maestroni, Francesco Bandello, Gianpaolo Zerbini

**Affiliations:** ^1^Department of Ophthalmology, Vita-Salute University, San Raffaele Scientific Institute, 20132 Milan, Italy; ^2^Complications of Diabetes Unit, Division of Metabolic and Cardiovascular Sciences, San Raffaele Scientific Institute, 20132 Milan, Italy

## Abstract

Although cellular and molecular bases of proliferative diabetic retinopathy are only partially understood, it is evident that this complication of diabetes is characterized by the formation of new vessels inside the retina showing abnormal architecture and permeability. This process, if not controlled by selective laser photocoagulation, leads to irreversible retinal damages and loss of vision. Angiogenesis, that is, the condition characterized by the growth of new blood vessels originated from preexisting ones, was shown to have a major role in the pathogenesis of proliferative retinopathy and, as a consequence, intravitreal antiangiogenic injection was suggested as a feasible treatment for this disease. Here, we describe the different antiangiogenic approaches used to treat this disease along with the respective advantages and limitations when compared to laser treatment. Altogether, even though further and longer studies are still needed to clarify the best possible therapeutic protocol, the antiangiogenic treatment will reasonably have a future role in the therapy and prevention of proliferative diabetic retinopathy.

## 1. Introduction

As a consequence of the ongoing worldwide epidemic of type 2 diabetes [1], we expect that in few years a similar outbreak of diabetic complications, and in particular of diabetic retinopathy, will eventually follow [2, 3]. Among the complications of diabetic retinopathy, which carry an important vision impairment, there are diabetic macular edema and proliferative diabetic retinopathy. More recent data comes from study conducted in USA in which investigators estimated that the prevalence of diabetic retinopathy was 28.5%, among persons with diabetes aged 40 years and older. Approximately, 1.5% of adults with diabetes had proliferative diabetic retinopathy and 2.7% had clinically significant macular edema [4].

In particular, the proliferative stage is characterized by the formation of new leaky vessels spreading without regular orientation on the retinal surface, often invading the vitreous cavity, and finally leading to hemorrhage, fibrosis, and tractional retinal detachment.

Despite the evidence that the prevalence of proliferative diabetic retinopathy (PDR) is progressively decreasing as a consequence of the improved techniques aimed to control glucose metabolism [4–7], the overall situation is worsening once again as a consequence of the increased prevalence of type 2 diabetes. A triplication of new cases of PDR is foreseen in the next forty years [2].

## 2. Angiogenesis

Angiogenesis is the physiologic condition characterized by the growth of new blood vessels originated from preexisting ones.

The angiogenic process follows several steps: first of all, a number of angiogenic growth factors activate the receptors present on resident endothelial cells. Once activated, the endothelial cells begin to release specific enzymes called proteases that degrade the basement membrane, finally allowing endothelial cells to leave the original (parental) vessel wall. At this stage, endothelial cells proliferate into the surrounding matrix, taking advantage of adhesion molecules called integrins. Angiogenesis may represent a pharmacological target for combating diseases characterized by either poor vascularization or hypertrophic vasculature. Antiangiogenic therapies, in particular, are presently employed to fight cancer and other malignancies.

Concerning the eye, the angiogenic process has to be considered as a pathologic phenomenon. There are actually several conditions leading to the formation of abnormal neovascularization. Age-related macular degeneration is one of the most important diseases characterized by the formation of choroidal new vessel in the macular region finally leading; if untreated, to vision loss. The other major disease characterized by abnormal formation of retinal vessels is diabetic retinopathy, in particular the so-called proliferative stage of this disease. Regarding both retina and choroid, vascular endothelial growth factor (VEGF) was shown to be a major contributor to angiogenesis by increasing the number of new capillaries.

VEGF concentration levels, in particular, were found to be significantly increased in ocular tissues from patients with diabetes [8]. This finding raised the question of the potential role of VEGF in the pathogenesis of DR.

## 3. Role of Angiogenesis in the Pathogenesis of PDR

A number of evidences suggest that VEGF, and consequently angiogenesis, is involved in the pathogenesis of PDR [9]. The finding that most of VEGF production in solid tumors is due to hypoxia stimulus [10] suggested that VEGF might be an ideal candidate to mediate the hypoxia-induced intraocular neovascular response. Furthermore, VEGF is both an endothelial specific mitogen and vascular permeability factor. This finding suggests that VEGF could account for both proliferation and vasopermeability in course of proliferative diabetic retinopathy [11].

In humans, diabetic patients with established PDR have indeed high levels of VEGF in the vitreous and this dysfunction can be normalized only by laser photocoagulation [12]. Accordingly, in mouse models of ischemic retinopathy, it is also possible to prevent the development of the proliferative stage by blocking VEGF activity [13]. Finally, intravitreal injection of VEGF was shown to cause iris neovascularization in primates [14].

From a functional point of view, VEGF has been identified as a proinflammatory mediator, reasonably involved in the development of the inflammatory process that accompanies the progression of DR. VEGF actually increases the expression of the cellular adhesion molecule ICAM-1 a chemotactic factor for monocyte/macrophage lineage cells [15, 16]. Through the activation of ICAM-1, VEGF, therefore, promotes leukostasis (and vascular leakage) and increases leukocyte counts in the retinas of diabetic animals [17, 18] and in human diabetic retinas [19] Conversely, blockage of VEGF decreases retinal leukocyte counts in experimental diabetes [20]. Altogether these findings provided a robust rationale for the setting up of clinical trials to verify VEGF blockade as a therapy for DR.

## 4. Intravitreal Anti-VEGF Treatment as a Therapy for PDR: Comparison with Laser Treatment

Panretinal photocoagulation (PRP) is at present the only successful evidence-based treatment for PDR. PRP reduces in fact the risk of severe visual loss by 50–60% with regression of the majority of neovascularizations over a period of 3 months. In particular, it was shown that when PDR regresses within the first 3 months after PRP treatment, the visual prognosis tends to be excellent [21].

Among the proposed mechanisms underlying PRP effectiveness are reduced oxygen requirement that follows the destruction of the highly metabolically active outer retinal cells and improved retinal oxygenation derived from choroidal circulation. Several attempts have been made to modify PRP laser techniques to reduce side-effects such as decreased visual acuity, peripheral field loss, and macular edema [22]. Despite this evidence, several patients still require supplemental laser treatment, and nearly 4.5% show disease progression that finally require pars plana vitrectomy (PPV), even in presence of an adequate PRP [23].

Limits of PRP include poor response to treatment, pain, nyctalopia, loss of peripheral vision, uveal effusions, worsening of macular edema, and difficulty to treat eyes with vitreous hemorrhage.

Most patients require at least two treatment sessions and several return for multiple additional sessions in case of persistent neovascularisation.

Altogether, nondestructive approaches alternative to PRP, such as VEGF inhibition, have been recently investigated as possible new therapies for PDR [24].

The molecules currently under investigation to treat PDR are Macugen (Pegaptanib sodium), Lucentis (Ranibizumab), and Avastin (Bevacizumab). The widespread use of these molecules in clinical practice is so far limited by their short-lived effects and the lack of established protocols.

### 4.1. Macugen

Pegaptanib sodium (Macugen, Eyetech Inc, Cedar Knolls, NJ, USA) is a 28-nucleotide RNA aptamer that binds specifically to the VEGF-A165 isomer, the major pathological VEGF protein in the eye.

### 4.2. Lucentis

Ranibizumab (Lucentis; Genentech USA, Inc., San Francisco, CA, USA/Novartis ophthalmics, Basel, Switzerland) is an engineered, humanized, recombinant antibody fragment (Fab) active against all VEGF-A isoforms. As it lacks the Fc domain, it has a much shorter half-life than other anti-VEGF agents. Lucentis is presently licensed as an intravitreal agent for the treatment of wet, age-related, macular degeneration (ARMD).

### 4.3. Avastin

Bevacizumab (Avastin; Genentech Inc., San Francisco, CA, USA) is a full-length recombinant humanized antibody active against all isoforms of VEGF-A. This large sized molecule (molecular weight: 148 kDa) has two times the half-life of ranibizumab, with a prolonged effect on retinal neovascularisation [25].

Bevacizumab is currently not licensed for intraocular use but is nonetheless the most used among anti-VEGF agents. Three randomized nonplacebo controlled trials on intravitreal bevacizumab for the treatment of PDR have been recently published [26–28]. Several other clinical trials are presently ongoing (http://www.clinicaltrials.org).

The standard average endpoint for evaluating the effectiveness of anti-VEGF treatments is commonly considered as the persistence of the effect of treatment for at least 6 months after the intraocular injection. Unfortunately, few clinical studies include a 6-month followup. Comparative analysis between different studies is not so simple as quantitative evaluation of the extent and severity of neovascularization differ between individuals. And this makes more difficult the translation of results of these studies into clinical practice.

In a retrospective analysis, Adamis et al. [29] demonstrated a persistent beneficial effect of intravitreal pegaptanib in patients with PDR, with 62% of the treated eyes showing regression or absence of neovascularization 6 months after injection.

A recent study from Cho et al. [30] studied the effects of intravitreal injection of Bevacizumab on VEGF expression and inflammation in fibrovascular membranes from 18 patients with PDR. An immunohistochemical staining for VEGF, CD31, and CD68 was performed in three different groups; group 1 : 4 inactive PDR eyes, group 2 : 10 active PDR eyes treated preoperatively with adjunctive intravitreal bevacizumab, group 3 : 5 active PDR eyes not treated preoperatively with bevacizumab. They found that IVB caused some reduction in VEGF expression and vascular densities in a limited number of active PDR patients, but they also demonstrated that a single injection may not be enough to induce complete blockage of VEGF and pathologic neovascularization in active PDR patients.

A possible solution to overcome these limits in efficacy could result from combined therapy consisting in laser treatment followed by intravitreal injection of anti-VEGF. This approach could have some advantage by increasing the extent of treatment, by accelerating the effect of laser photocoagulation, and by providing alternative therapeutic solutions when laser delivery by itself is difficult or impossible.

## 5. Bevacizumab

Concerning Bevacizumab and laser treatment, the study done by Mirshahi et al. [31] is probably the largest performed to date. Forty patients with type 2 diabetes and PDR in both eyes with high risk profile underwent scatter laser treatment following the ETDRS protocol and had a single bevacizumab injection in one eye; sham injection was performed in the controlateral eye used as control. This study demonstrated that at week six 87.5% of eyes treated with bevacizumab had complete regression of neovascularization versus 25% in the sham treated group (*P* < 0.005). At week 16, the difference between the two groups disappeared. This study provides further evidence that bevacizumab has an inhibitory effect on the formation of new vessels. This study allows to conclude that intravitreal bevacizumab is a valid treatment for early high-risk PDR.

### 5.1. Eyes Resistant to Panretinal Photocoagulation (PRP)

The effect of intravitreal Bevacizumab in eyes with persistent, active PDR was assessed by Jorge and colleagues in a noncomparative trial [32]. One injection of bevacizumab was administered to 15 eyes that were then followed for 12 weeks. As a result, best-corrected visual acuity (BCVA) was improved significantly from baseline at all time points (1, 6, and 12 weeks), from 20/160 at baseline to approximately 20/125 at 12 weeks. The mean area of fluorescein leakage was also improved significantly at all time points. No significant adverse events could be demonstrated. If these results will be confirmed by further and larger studies, bevacizumab will be identified as an important intervention for eyes with refractory PDR.

Intravitreal bevacizumab for cases that were not responsive to traditional PRP has been evaluated in another study by Moradian and colleagues [33]. Thirty eight eyes received a bevacizumab injection at baseline, and after 6 or 12 weeks according to the research protocol. Clearance of vitreous hemorrhage and regression of active fibrovascular tissue were considered as endpoints. A tendency toward resolution of vitreous hemorrhage with a trend toward significance could be shown at 6 weeks (*P* = 0.06). No significant change in the extent of fibrovascular tissue occurred, even though several eyes could not be evaluated for this variable because of media opacity. The most remarkable finding in this study was probably the occurrence of two tractional retinal detachments (5.3% of study eyes). This finding is in line with the report by Arevalo and colleagues that 5.2% of the eyes with PDR developed TRD after an extra intravitreal bevacizumab injection performed before vitrectomy [34].

Taken together, the above-described studies suggest that intravitreal bevacizumab decreases leakage from diabetic neovascular lesions in newly diagnosed and refractory disease. Further studies are now necessary, particularly on possible long-time side effects before we will be able to translate these research findings into clinical practice.

### 5.2. In Case of Vitreous Hemorrhage

Persistent and recurrent vitreous hemorrhage is a common complication of vitrectomy for diabetic retinopathy with an incidence ranging from 12% to 63% [35].

Bevacizumab was shown to reduce intra- and postoperative bleeding and surgical operating times when used before the surgical removal of vitreomacular membranes [36–38] In most studies, bevacizumab has been administered for just one week preoperatively, to avoid the occurrence of tractional retinal detachment in patients with severe PDR [33, 34].

In a study, Rizzo and colleagues randomized 22 eyes with severe PDR and TRD either to intravitreal bevacizumab 5 to 7 days before PPV or to placebo [28]. As a result, they demonstrated that difficulties in the surgical procedure, as evaluated by recording operative times, number of instrument exchanges, number and severity of intraoperative bleeds, dissection techniques, and intraoperative retinal tears were reduced in the bevacizumab group.

Similarly, Yeh and colleagues tested the effect of bevacizumab as an adjuvant therapy 1 week before vitrectomy [37]. They enrolled 41 eyes with severe PDR and active fibrovascular proliferation extended to the periphery. The authors randomized these eyes to bevacizumab or to placebo. As a result, intraoperative bleeding from proliferative tissue was significantly worse in the control group, even though intraoperative subretinal hemorrhage was more frequent in the bevacizumab group (*P* = 0.004). The authors concluded that although an increased rate of intraoperative subretinal hemorrhage occurred in the bevacizumab group, several potential benefits of the drug finally outweighed the observed adverse effects.

A large trial of bevacizumab along with vitrectomy has been recently performed by Ahmadieh and colleagues [38]. The authors randomized 68 eyes scheduled to undergo PPV for PDR to intravitreal bevacizumab, 1 week before PPV, or to sham injection. Only 34 eyes completed the study as in several cases treated with bevacizumab a significant improvement during the week after the injection could be demonstrated. The incidence of postvitrectomy haemorrhage 1 week and at 1 month after surgery was significantly lower in the group treated with bevacizumab compared to the controls (*P* = 0.023 and *P* = 0.001, resp.). Also intraoperative bleeding was significantly less in the bevacizumab group (*P* = 0.035), as was the need to use intraoperative endodiathermy.

Altogether, the above-described studies indicate that bevacizumab before vitrectomy represent a valid approach for PDR. Injection of bevacizumab 1 or 2 weeks before PPV did not cause any adverse outcomes. Further studies with a larger number of patients are now warranted to confirm these preliminary results.

### 5.3. In Case of Neovascular Glaucoma

Anti-VEGF agents might have role in the management of one of the most severe forms of secondary glaucoma, the so-called neovascular glaucoma (NVG).

On this regard, Chalam et al. [39] reported complete regression of neovascularization due to aggressive NVG within 3 weeks from the treatment with bevacizumab.

A trial on 26 eyes with NVG was performed by Costagliola et al. [40]. The authors demonstrated that at the end of the treatment, in all patients, it was possible to appreciate a regression of neovascularisation paralleled by a reduction of intraocular pressure (IOP). After one year of followup, however, three eyes required glaucoma valve implants and 14 patients were treated with standard glaucoma medication.

A massive regression of iris neovascularization in a 2-week period and no significant changes in IOP could be demonstrated in NVG patients treated with injection of bevacizumab by Lim et al. [41].

Finally, Eid et al. [42] recently demonstrated that combining bevacizumab with good PRP ablated the ischaemic retina and ensured good success rates in 20 patients with intractable glaucoma.

### 5.4. In Case of Chataract Surgery

Sixty-eight eyes with any type of DR at the end of cataract surgery were randomized to bevacizumab by Cheema and colleagues [43]. As a result, 1 month after treatment, 5 control eyes progressed in the severity of DR versus only four treated eyes (*P* = 0.002). Macular edema was also more common in control eyes.

Takamura and colleagues also injected bevacizumab at the conclusion of cataract surgery in diabetic patients [44]. During the followup the treated eyes, when compared to control eyes had a significant improvement with respect to preoperative measurements.

A similar study was performed by Lanzagorta-Aresti and colleagues [45] in patients with moderate NPDR and DME. Twenty-six eyes that underwent laser treatment followed by uncomplicated cataract surgery received bevacizumab or sham injection. As a result, the treated group showed a significant improvement in BCVA and no change in CMT. The sham group showed a worsening of visual acuity and a significant increase in CMT. Although the results look promising, further studies are now necessary to confirm these early findings.

## 6. Pegaptanib Sodium

The effect of intravitreal Pegaptanib (Macugen) on diabetic macular edema [46] was evaluated in retrospective analysis aimed to compare the effect of pegaptanib on ocular neovascularization to a sham group. Sixteen subjects were included in the study. Eight subjects in the intravitreal pegaptanib group (*n* = 13) showed regression of neovascularization (62%) at 36 weeks, whereas none of the eyes in sham group (*n* = 3) showed regression of neovascularization. However, in three of the eight treated eyes (37.5%), ocular neovascularisation recurred at the end of followup.

More recently, González et al. [47] performed a prospective, randomized, controlled, open label study aimed to clarify the efficacy of intravitreal pegaptanib versus PRP in the treatment of active PDR. As a result, by week 12, in all eyes receiving pegapanib, a complete regression of retinal proliferation could be demonstrated and was maintained through week 36.

## 7. Ranibizumab

There are no final reports on the effect of ranibizumab on PDR [48]. The Diabetic Retinopathy Clinical Research Network (DRCRnet) is presently performing a randomized prospective controlled trial to determine whether intravitreal ranibizumab or a steroid given to patients with PDR and macular edema can reduce the risk of visual loss following PRP and provide good visual outcomes over a short term. The primary outcome measure includes visual acuity outcomes at 14 weeks. Secondary outcome measures include changes in retinal thickness, presence, and extent of new vessels on fundus photos and vitreous haemorrhage.

The study is currently closed and the scientific community is waiting for the final results. (Intravitreal Ranibizumab or Triamcinolone Acetonide as Adjunctive Treatment to Panretinal Photocoagulation for Proliferative Diabetic Retinopathy. Available at: http://drcrnet.jaeb.org/ Accessed: May 10, 2011). 

## 8. Other Anti-VEGF Drugs

Among the others, a new and alternative way to block VEGF is represented by VEGF-trap (aflibercept). VEGF-trap is a fusion protein made of immunoglobulin domains of both VEGF-1 and -2 fused to an Fc-fragment of human IgG. VEGF-trap acts as a soluble receptor as it is able to bind every isoform of extracellular VEGF [49]. Whether this approach is really effective and may reduce the side effects of standard anti-VEGF therapy remains to be seen. A major problem with pan-isoform blockade of VEGF is indeed the decrease in physiologic revascularization, a process that is important in preventing PDR [50].

RNA interference is a classic example of basic research that has moved from bench to bedside. Intracellular transcription of VEGF can actually be shut down by means of RNA interference, finally decreasing the production of VEGF released from the retinal pigment epithelium. This kind of approach is presently studied in treatment of wet AMD [51]. As previously done with other anti-VEGF drugs, after demonstration of its safety and efficacy in neovascular AMD, RNA interference will for sure explore also patients with PDR.

Finally, another novel therapy may consist in the use of small molecules that, acting as tyrosine kinase inhibitors, become able to inhibit the intracellular signaling cascade of VEGF. These substances could be of use in the treatment of PDR [52], although the results of preliminary studies seem to suggest the exacerbation of diabetic neuropathy as a possible, not irrelevant, side effect [53].

There are presently just few studies aimed to evaluate the above-described new drugs in the treatment of diabetic retinopathy, and they are all referred to patients with diabetic macular edema.

## 9. Limits of Anti-VEGF Treatment

A major limit in anti VEGF treatment consists in the evidence that recurrence of retinal neovascularisation following anti-VEGF treatment is a quite common finding in a period that ranges between 2 weeks [54] to 3 months [55, 56], after injection. A reinjection 3-month after the baseline is probably a reasonable timing in most cases, especially in case of patients with high-risk PDR. Results in this field are still discrepant between different groups. Minnella reported that the effects of bevacizumab were maintained at 3 months in 15 treated eyes [57].

Conversely, Schmidinger et al. [58] reported that 62% (8 of 13) of treated eyes required retreatment with bevacizumab 3 months after baseline injection because of the appearance of new vessels.

## 10. Side Effects of Anti-VEGF Treatment

Along with its therapeutic effect on ocular neovascularization, Bevacizumab treatment may be accompanied by a number of side effects. Tractional retinal detachment (TRD) may sometime affect patients with severe PDR [59] treated with this drug. It has been hypothesized that bevacizumab might induce a fibrotic occlusion of new vessels. The contraction of this fibrous tissue may, therefore, result in TRD and vitreous haemorrhage [60–62]. Alternative mechanisms underlying the development of TRD could be the high fluctuations in intraocular pressure (IOP) [63] and deformation of the eye during intravitreal injection with possible intrusion of the vitreous in the sclera, resulting in vitreoretinal traction [64]. A possible explanation for the increased IOP could be the blockage of the internal trabeculae by bevacizumab itself that, being a large 148-kDa protein, may act as an additional barrier [65].

Lee and Koh [66] documented angiographically a foveal avascular zone enlargement following pars plana vitrectomy and treatment with bevacizumab. The authors attributed this finding to a total, nonselective blockage of VEGF levels, when it is well established that physiological concentrations of VEGF are thought to be essential for maintaining foveal circulation and visual acuity.

Further studies are needed to verify the systemic side-effects of anti-VEGF agents, particularly in diabetic subjects with significant vascular complications. Among the systemic side effects, the most common is hypertension (5.6%), followed by other cardiovascular complications [67, 68]. The use of bevacizumab in women of child bearing age need to be carefully monitored Kumar et al. [69].

At the moment, a large prospective trial aimed to verify the presence of short- and long-term adverse effects of bevacizumab treatment is still lacking.

The largest dataset for bevacizumab treatment is presently represented by a retrospective study [70] of 1,173 patients who received intravitreal bevacizumab and were followed for 12 months. A number of adverse effect were reported: seven cases of acute elevation of blood pressure, six strokes, five myocardial infarctions, five deaths, seven cases of bacterial endophthalmitis, seven cases of tractional retinal detachment, and four cases of uveitis.

Mason and colleagues retrospectively studied 5,233 intravitreal bevacizumab treatments and found a single case of acute postinjection endophthalmitis [71]. Safety concerns the use of bevacizumab comes from studies of the intravenous use in cancer therapy. Established side-effects in these studies include arterial thromboembolism, gastrointestinal perforation, hemorrhage, hypertensive crisis, and nephrotic syndrome [72, 73]. Concerning other anti-VEGF treatments, the VISION trial performed in patients with neovascular AMD treated with intravitreal pegaptanib [74, 75] reported no systemic side effects that could be attributed to treatment over the course of the study. Some rare specific ocular complications, such as endophthalmitis, traumatic lens injury, or retinal detachment, were attributed to the injection procedure rather than to the medication.

The MARINA and ANCHOR studies aimed to treat neovascular AMD, reported the safety of intravitreal ranibizumab. The MARINA study (two-year observation) showed no increase in systemic adverse effects with ranibizumab [76]. By pooling together the safety data from PIER, MARINA, and ANCHOR (one-year observation) it was possible to demonstrate an increased rate of vascular events (2.1% rate of myocardial infarction and stroke) in the ranibizumab arms versus the control (1.1%) [77].

Finally, although VEGF has been implicated in the development of a number of ocular neovascular diseases, physiologic concentrations of endogenous VEGF play a strong role not only in maintaining the correct perfusion of the retina, but they also have a key role in the survival of the retinal neuron, the Muller cell, and photoreceptors [78, 79].

A recent study conducted in mouse eyes, in fact, reported a significant loss of neuronal retinal ganglions cells due to a chronic inhibition of VEGF [80]. Caution must be warranted.
